# Metabolism-Associated Molecular Classification of Colorectal Cancer

**DOI:** 10.3389/fonc.2020.602498

**Published:** 2020-12-04

**Authors:** Meng Zhang, Hai-zhou Wang, Ru-yi Peng, Fei Xu, Fan Wang, Qiu Zhao

**Affiliations:** ^1^ Department of Gastroenterology, Zhongnan Hospital of Wuhan University, Wuhan, China; ^2^ Hubei Clinical Center & Key Lab of Intestinal & Colorectal Diseases, Wuhan University, Wuhan, China

**Keywords:** colorectal cancer, classification, metabolism, immune signatures, non-negative matrix factorization

## Abstract

The high heterogeneity of colorectal cancer (CRC) is the main clinical challenge for individualized therapies. Molecular classification will contribute to drug discovery and personalized management optimizing. Here, we aimed to characterize the molecular features of CRC by a classification system based on metabolic gene expression profiles. 435 CRC samples from the Genomic Data Commons data portal were chosen as training set while 566 sample in GSE39582 were selected as testing set. Then, a non-negative matrix factorization clustering was performed, and three subclasses of CRC (C1, C2, and C3) were identified in both training set and testing set. Results showed that subclass C1 displayed high metabolic activity and good prognosis. Subclass C2 was associated with low metabolic activities and displayed high immune signatures as well as high expression of immune checkpoint genes. C2 had the worst prognosis among the three subtypes. Subclass C3 displayed intermediate metabolic activity, high gene mutation numbers and good prognosis. Finally, a 27-gene metabolism-related signature was identified for prognosis prediction. Our works deepened the understanding of metabolic hallmarks of CRC, and provided valuable information for “multi-molecular” based personalized therapies.

## Introduction

Colorectal cancer (CRC) is one of the most frequently diagnosed cancers all around the world. There are over 1.8 million new cases and almost 900,000 deaths annually (1, 2). Although new treatment options, such as targeted therapy and immunotherapy, have been developed, the average 5-year survival probability for advanced CRC patients is still dismal, lower than 15% (3, 4). What’s worse is that the incidence of CRC in patients who are younger than 50 years is rising sharply, and the mortality of CRC has ranked the first for men in age 20–49 during 2012 to 2016 (5, 6). As we were known, CRC is a heterogeneous disease, therefore, more researches should be conducted for precisely understanding the molecular properties of CRC (7).

Besides the classic TNM staging based on histopathology, CRC also has several molecular traits, such as chromosomal instability (CIN), microsatellite instability (MSI) and CpG island methylator phenotype (CIMP) (8). With the accumulation of multiple kinds of “omics” data, CRC samples have been classified into four consensus molecular subtypes (CMS) in 2015, including CMS1 (MSI Immune, 14%), CMS2 (Canonical, 37%), CMS3 (Metabolic, 13%), and CMS4 (Mesenchymal, 23%). The CMS groups had distinct characteristics, which contributed to targeted interventions for CRC patients. For example, KRAS mutations were overrepresented in CMS3, therefore, epidermal growth factor receptor (EGFR) antibodies should be avoided for these CRC patients (9, 10). More recently, some signatures, especially immune alterations, were utilized for molecular subtyping in many kinds of cancers. Three immune subtypes were identified and validated in lower-grade diffuse glioma, and they were characterized with different lymphocyte signatures, somatic DNA alterations and clinical outcomes (11). Microsatellite instability-high (MSI-H) CRC patients were separated into two different subtypes by consensus clustering, which showed distinct molecular profiles (12).

Metabolism reprogramming is one of the hallmarks of cancer (13). In order to meet the growing demands for energy requirement for cell proliferation, tumor cells owned unique metabolic way of glucose, glutamine, fatty acids, amino acid and many other kinds of nutrients and metabolites, such as aerobic glycolysis, *de novo* synthesis of fatty acids (14). Nowadays, targeting the metabolic differences between tumor and normal cells have become a promising anticancer strategy. Moreover, a deeply exploring of molecular changes induced by metabolism rewiring can contribute to the development of targeted therapies (15). Recently, a study had classified hepatocellular carcinoma (HCC) samples into three subclasses based on a panel of metabolic genes, including active (C1), intermediate (C2), and exhausted (C3) metabolic subtype. Each subtype had distinct molecular, immune and clinical features. For instance, C1 had the best prognosis and matched the characteristics of non-proliferative HCCs. C2 exhibited high immune infiltration and sensitivity toward immune blockade as well as chemotherapy. What’s more, a meaningful 90-gene classifier was provided, which may help to predict the prognosis of HCC patients and prospective therapies (16). However, research on metabolism-relevant molecular classification of CRC has not yet been reported.

In the present study, a non-negative matrix factorization (NMF) clustering based on metabolic genes was performed and validated in CRC datasets. Three distinct subtypes were identified, namely C1, C2, and C3. Then, we revealed the prognosis traits, metabolic signatures, transcriptome features, clinical characteristics, immune infiltration as well as gene mutation alterations among the three subclasses. Furthermore, a metabolism-related signature was also identified and validated.

## Methods

### Data Source and Processing

The CRC clinical and molecular data (including RNA expression and mutation) were extracted from the Genomic Data Commons (GDC) Data Portal (https://portal.gdc.cancer.gov/). Normal samples, repeated samples and samples without key clinical features were excluded for further analyses. After procession, there were 435 patients in GDC TCGA COAD project included in training study. 375 of the above 435 patients had mutation data. For validation, the human CRC mRNA expressing data were downloaded from Gene Expression Omnibus (GEO) database (http://www.ncbi.nlm.nih.gov/geo/). Dataset GSE39582, containing 585 CRC samples, was chosen as testing set, and 556 of which were finally selected after data filtration.

### Identification of CRC Subclasses

In the present study, we prepared a total of 2,752 metabolism-related genes involved in all metabolic process for non-negative matrix factorization (NMF) clustering (17). Before classification, a filtering procedure was conducted. Firstly, some candidate genes, whose expression value was zero in any analyzed sample and whose median absolute deviation (MAD) value was lower than 0.5 across all the samples, were excluded. Next, Cox proportional hazards model was conducted by “survival” R package to screen meaningful genes for overall survival (OS). Finally, metabolism-associated genes with relatively high variable (MAD > 0.5) and significant prognostic value (P < 0.05) were chosen for subsequent clustering analysis. The way of unsupervised NMF clustering was implemented by “NMF” R package on the training and testing datasets (18). The corresponding codes were provided in supplementary methods. The value was determined by the cophenetic correlation coefficient, the magnitude of which began to fall was chosen as the optimal number of clusters (19). Principal components analysis (PCA) was used to access expression differences between the subtypes.

### Gene Set Variation Analysis

Gene set variation analysis (GSVA), a nonparametric and unsupervised gene set enrichment method, can calculate the score of a certain pathway or a signature based on transcriptomic data (20). We acquired the 115 metabolism-associated gene signatures from previously published works (21). Several CRC progression relevant signatures were also downloaded from the Kyoto Encyclopedia of Genes and Genomes (KEGG) database. Then, each sample got a score corresponding to the above signatures by “GSVA” R package. Utilizing “limma” R package, differential analyses were subsequently conducted based on the signature cores, and the signatures with an absolute log2 fold change (FC) > 0.4 (adjusted P < 0.05) were defined as significant differentially expressed signatures. The results were visualized by using “ComplexHeatmap” R package.

### Differentially Expressed Gene and Gene Ontology Analyses of CRC Subclasses

The “limma” R package was also utilized to calculate the DEGs among CRC subclasses. Adjusted P value < 0.05 and |log2FC| > 0.5 were set to choose significant DEGs. Then, GO enrichment analysis and visualization were performed *via* “clusterProfiler” R package (22).

### Immune Infiltration Estimation and Immunotherapy Prediction of CRC Subclasses

Firstly, the immune score, stromal score and tumor purity were calculated by the ESTIMATE algorithm, which can reflect the enrichment of stromal and immune cell gene signatures (23). Then, the online CIBERSORT method (https://cibersortx.stanford.edu/) was used to evaluate the LM22 gene signatures in CRC subtypes (24). Furthermore, the other signature contained 17 immune cell types was provided, and the single-sample GSEA (ssGSEA) algorithm was applied to estimate the immune infiltration. Differential analyses were conducted as described above, and data were visualized by the heatmap. The expression data from melanoma patients treated with immunotherapies were extracted from the published work (25). SubMap analysis (Gene Pattern) was applied to compare the correlation of gene expression profiles between our subclasses and melanoma patients.

### Mutation Differences of CRC Subclasses

The MAF files contained the mutation information of training set were downloaded and processed. The “maftools” R package was utilized to analyze gene mutations among CRC subclasses (26).

### Metabolism-Related Signature Construction

LASSO penalized Cox regression model was built by “glmnet” R package (27), and the lambda.1se, a penalty parameter for prevention of overfitting, was selected to construct an optimal and prognostic gene set. Finally, the risk scores of each samples was calculated by the formula: $\text{Risk}\;\text{score}\;=\Sigma_{i=1}^{N}\;Exp_{i}*\beta_{i}$.

### Statistical Analysis

Survival analyses were performed by Kaplan–Meier methods and compared by the log-rank test. ROC curve was analyzed, and the area under the curve (AUC) was calculated using the ‘‘survivalROC’’ package. The relationship between CRC subclasses and the clinical features was estimated by Chi-square analysis. Unpaired Student’s t-test was used to compare two groups with normally distributed variables, one-way analysis of variance was used for three group comparison. A two-tailed P value < 0.05 was statistically significant.

## Results

### NMF Identifies Three Metabolism Subclasses in CRC

**Figure 1:**
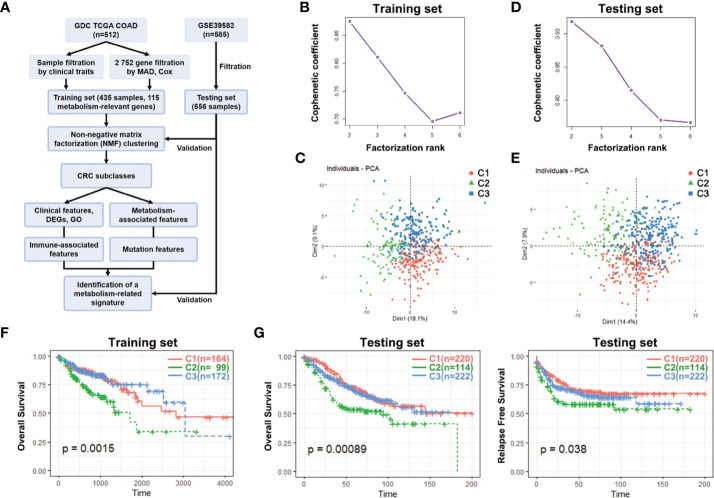
Identification of CRC subclasses using NMF consensus clustering. **(A)** A flow chart of the study. **(B)** NMF clustering using 115 metabolism-associated genes in training set. Cophenetic correlation coefficient for k = 2–6 is shown. **(C)** PCA showed the distribution of three CRC subclasses in training set. **(D)** Cophenetic correlation coefficient in testing set. **(E)** The distribution of three CRC subclasses in testing set. **(F)** OS of three subclasses (C1, C2, and C3) in training set. **(G)** OS and RFS of three subclasses in testing set. CRC, Colorectal cancer; NMF, Non-negative matrix factorization; MAD, Median absolute deviation; PCA, Principal components analysis; OS, Overall survival; RFS, Relapse free survival; DEGs, Differentially expressed genes; GO, gene ontology.

First of all, a flow chart was shown to introduce this study design (**Figure 1A**). Clinical characteristics of training set and testing set were listed in **Table 1**, and there was no significant difference in general features between two datasets. The training set had 435 valid CRC samples with complete clinical traits. For clustering, the mRNA expression matrix of the initial 2,752 metabolism-relevant genes in training set was acquired. After primary filtering, 1,514 genes were excluded for undetectable expression or low MAD, and 1,238 genes were selected for subsequent analysis. To get the metabolic genes with prognostic value for classification, univariate cox proportional hazards model was conducted. Results showed that only 115 of the above metabolic genes had significant risks on survival of patients in training set (**Table S1**). Moreover, multiple permutation testing was performed to confirm the robustness of the selected genes for classification (**Figure S1**) (28, 29). Therefore, a total of 115 genes were identified for NMF clustering. GO analysis showed that the 115 genes were mostly enriched in small molecule, oxoacid, organophosphate, lipid and some other metabolite metabolic process (**Figure S2A**). To find the optimal k value, cophenetic correlation coefficients were calculated. Data showed that the cophenetic correlation coefficient fell sharply when k = 3 (**Figure 1B**). Moreover, the consensus matrix heatmap also kept crisp boundaries at k = 3 (**Figure S2B**). Therefore, k = 3 was chosen as the optimal number of clusters. Namely, three clusters were identified in training set. There were 164 samples in the cluster 1 (C1), 99 in the C2 and 172 in the C3. To access the subclasses’ assignments, we performed PCA. Data showed that the three clusters were distributed in different corners of the two dimensional coordinate systems (**Figure 1C**). Furthermore, we extracted the expression data of the above selected 115 genes in testing dataset with 556 eligible CRC samples from GEO database (GSE39582). A similar NMF consensus clustering was performed. Consistently, the optimal K value was also 3 in testing set and three distinct subclasses were identified, which also showed the same distribution as that in training set by PCA (**Figures 1D, E** **, and** **S1C**).

**Table 1:** Clinical characteristics of training and testing sets.

Clinical characteristics		Training set (n=435) n(%)	Testing set (n=556) n(%)	Chi-square	P value
Gender	male	233(53.6)	306(55.0)	0.213	0.653
	female	202(46.4)	250(45.0)		
Age	<65	171(39.3)	210(37.8)	0.245	0.645
	≥65	264(60.7)	346(62.2)		
TNM stage	1–2	239(55.0)	295(53.1)	1.063	0.332
	3–4	185(42.5)	261(46.9)		
	*NA*	11(2.5)	0(0.0)		

To explore the differences among the three subclasses, the survival analyses were firstly performed. In the training set, the C2 had the shortest median survival time (MST) while the C3 had the longest. The OS probability within the three subclasses had significant differences (p = 0.0015) (**Figure 1F**). What’s more, the OS probability levels of the three subclasses in testing set had the same tendency as that in training set. The OS probability and relapse free survival (RFS) probability of the C2 was the lowest, and both had significant differences (p = 0.00089 for OS, p = 0.038 for RFS) (**Figure 1G**). These results demonstrated that the three subclasses had obviously different prognosis.

### Correlation of the CRC Subclasses With Metabolism-Associated Signatures

**Figure 2:**
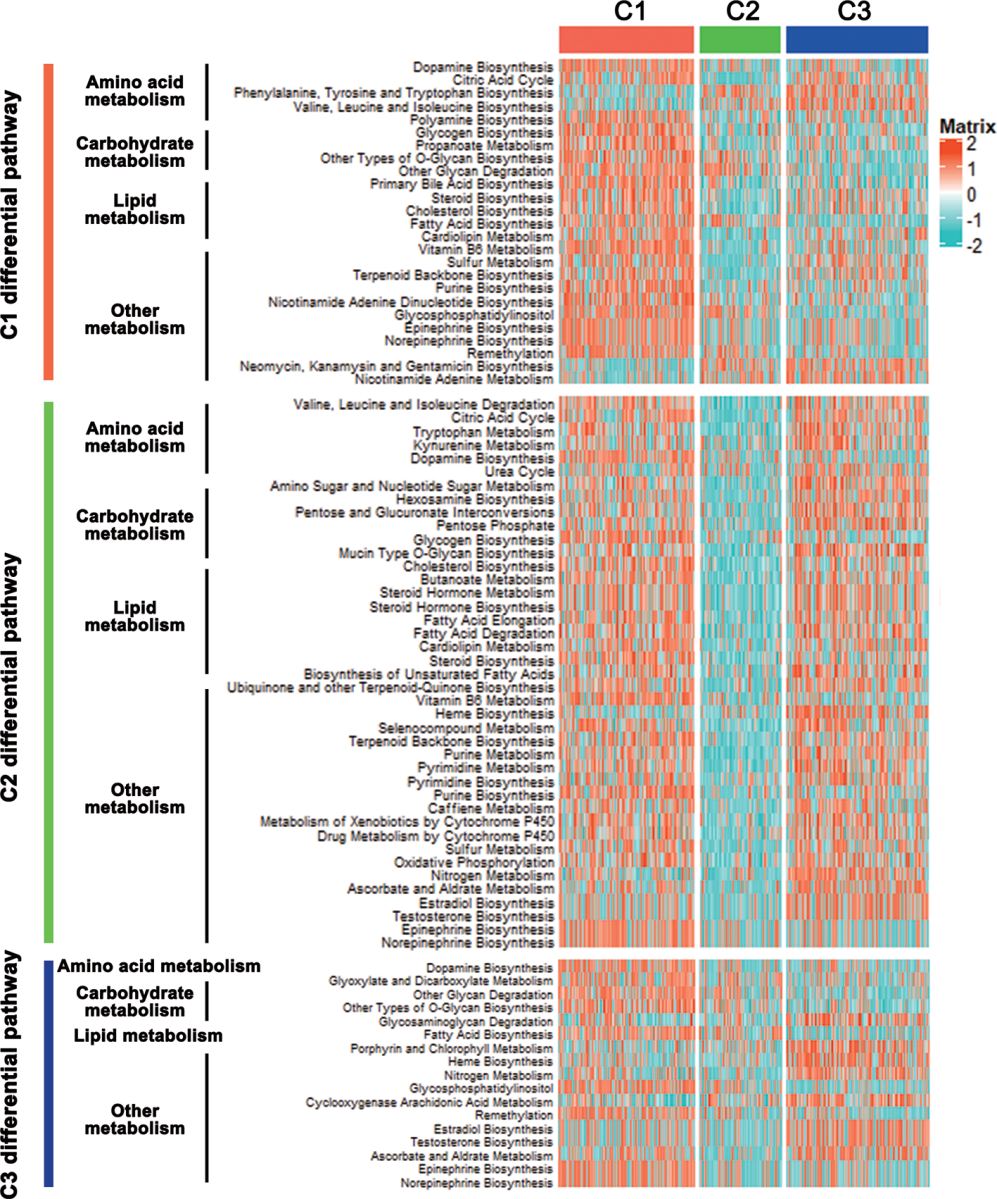
Differential metabolic pathways among the CRC subclasses. Heatmap of the significantly differential metabolic pathways of CRC subtypes in training set was shown.

**Figure 3:**
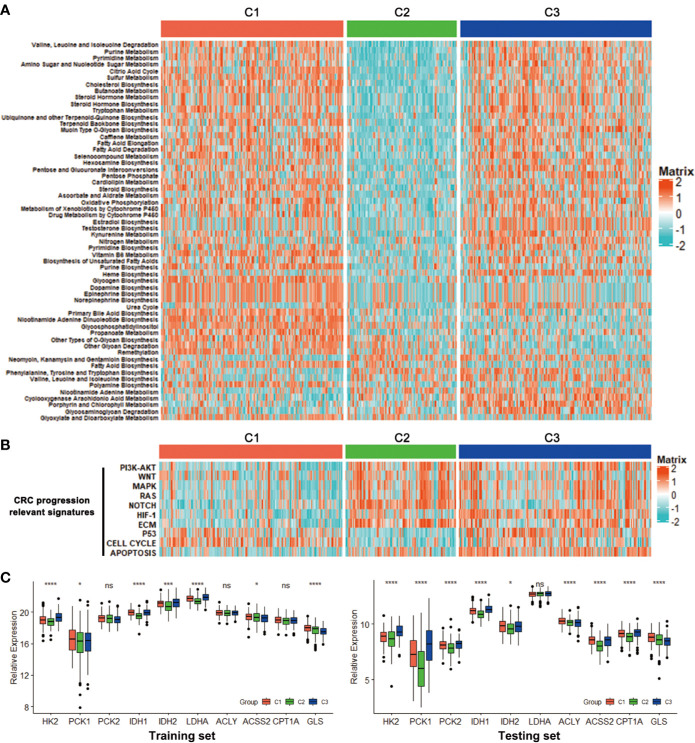
Association with metabolism and progression-associated signatures among the CRC subclasses. **(A)** Heatmap of the specific metabolism-associated signatures of CRC subtypes in training set. **(B)** Heatmap of the CRC progression relevant signatures in training set. **(C)** Expression differences of several key metabolic genes among three subclasses in training and testing sets. *P < 0.05, ***P < 0.001, ****P < 0.0001; ns, no significance.

The CRC classification was based on metabolism-relevant genes, therefore, we further studied whether distinct subclasses in training set had different metabolic characteristics. Firstly, 115 metabolism processes were listed and quantified by GSVA R package (**Table S2**). Each sample got a score for the correspondingly metabolic pathway. Then, differential analyses were performed to dig out subtype-specific metabolism signatures (**Figure 2**). Compared with C2 and C3, results showed that C1 had 25 kinds of significantly differential metabolic pathways, 5 of them were related to amino acid metabolism while 5 belonged to lipid metabolism. At the same time, most differential metabolism pathways were enriched in C1. Compared with C1 and C3, C2 had 41 kinds of significantly differential metabolic pathways, but all of which were downregulated, including amino acid, carbohydrate, lipid and other metabolism-related signatures. Moreover, there were 17 kinds of differential metabolic pathways in C3. Some other metabolism pathways, such as porphyrin and chlorophyll metabolism, heme biosynthesis, related to metabolism of cofactors and vitamins, were enriched in this subtype (**Figure 2**, **Table S3**). After merging duplicate pathways in three subtypes, a total of 58 metabolism**-**associated signatures were shown by a heatmap (**Figure 3A**). It clearly showed that C1 was metabolic active while C2 was metabolic exhausted, and C3 displayed intermediate activity. The above data demonstrated that the three subclasses were enriched with diverse metabolism pathways and had different levels of metabolic activity.

For further investigation, several CRC progression relevant pathways were also evaluated. Results exhibited that C1 had significantly higher Cell cycle signature than C2 and C3, and C2 displayed higher expression for PI3K-AKT, WNT, MAPK, RAS, NOTCH and ECM pathways, while C3 was especially enriched with HIF-1, P53 and Apoptosis pathways (**Figure 3B**). Moreover, the expressions of some key genes participated in glucose, fatty acid and glutamine metabolic process were analyzed. Data showed that C1 and C3 harbored a higher expression of these key metabolic genes, which was consistent with the metabolic pathway results (**Figure 3C**).

### Clinical Characteristics and DEGs of the CRC Subclasses

To better clarify the three CRC subclasses, the relationship with clinical features was studied by Chi-square test. The results in training set was shown in **Table 2**, which demonstrated that the proportion of samples in “TNM stage”, “T stage”, “N stage” and “M stage” were significantly different within distinct subtypes. Consistently, the difference of “T stage” and “M stage” within distinct subtypes of testing set also had significance. However, “TNM stage” and “N stage” had no significance. Furthermore, subtypes in testing set had significantly diverse proportion of “TP53 mutation”, “KRAS mutation” and “BRAF mutation” (**Table 3**).

**Table 2:** Clinical Characteristics of patients with distinct classification in training set.

Clinical characteristics		Total	C1	C2	C3	Chi-square	*P* value
		n = 435	n = 164	n = 99	n = 172		
Gender	male	233	83	58	92	1.58	0.454
	female	202	81	41	80		
Age	<65	171	67	40	64	0.532	0.767
	≥65	264	97	59	108		
TNM stage	1–2	239	89	38	112	18.399	<0.0001***
	3–4	185	69	59	57		
	*NA*	11	6	2	3		
T stage	Tis–T2	87	42	10	35	9.302	0.01*
	T3–T4	348	122	89	137		
N stage	N0	255	96	43	116	16.122	0.003**
	N1	102	42	30	30		
	N2	78	26	26	26		
M stage	M0	320	114	66	140	17.651	0.001**
	M1	61	25	23	13		
	MX	47	24	7	16		
	*NA*	7	1	3	3		

*P < 0.05, **P < 0.01, ***P < 0.001. NA, Not Available.

**Table 3:** Clinical Characteristics of patients with distinct classification in testing set.

Clinical characteristics		Total	C1	C2	C3	Chi-square	*P* value
		n = 556	n = 220	n = 114	n = 222		
Gender	male	306	113	62	131	2.634	0.268
	female	250	107	52	91		
Age	<65	210	84	39	87	0.821	0.663
	≥65	346	136	75	135		
TNM stage	1–2	295	123	50	122	4.911	0.086
	3–4	261	97	64	100		
T stage	Tis–T2	57	28	1	28	13.239	0.001**
	T3–T4	479	185	106	188		
	*NA*	20	7	7	6		
N stage	N0	296	124	51	121	9.36	0.053
	N1	131	53	23	55		
	NX	109	36	33	40		
	*NA*	20	7	7	6		
M stage	M0	473	194	84	195	12.298	0.002**
	M1–MX	63	19	23	21		
	*NA*	20	7	7	6		
TP53 Mutation	M	188	89	34	65	7.931	0.019*
	WT	156	51	32	73		
	*NA*	212	80	48	84		
KRAS Mutation	M	213	72	39	102	8.307	0.016*
	WT	322	136	72	114		
	*NA*	21	12	3	6		
BRAF Mutation	M	49	2	23	24	34.24	<0.0001***
	WT	453	192	85	176		
	*NA*	54	26	6	22		

*P < 0.05, **P < 0.01, ***P < 0.001. M, Mutation; WT, Wild Type.

NA, Not Available.

**Figure 4:**
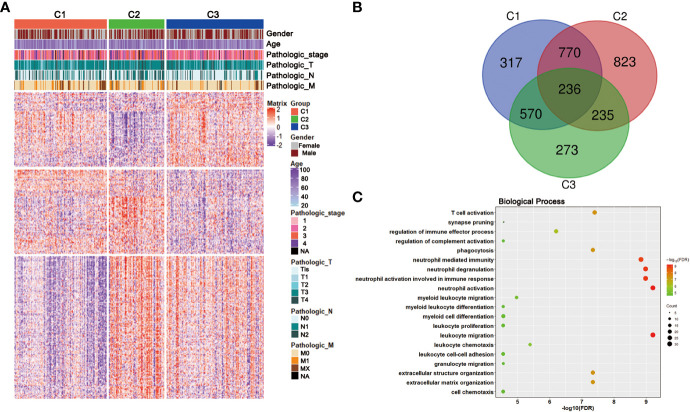
DEGs and GO analysis in the CRC subclasses. **(A)** DEG heatmap of CRC subtypes in training set, annotated by clinical traits. **(B)** Venn diagram showed the number of DEGs among three subtypes in training set. **(C)** GO results of the subclass-specific genes in training set.

To gain deeper insights into the molecular characteristics of the three CRC subclasses, the DEGs and their GO analysis were identified in training dataset. Under a threshold of Adjusted P value < 0.05 and |log2FC| > 0.5, a total of 5 271 DEGs were identified for the three subclasses. In detail, 1 893 DEGs were obtained for C1 compared with C2 and C3, 2,064 DEGs for C2 while 1,314 genes for C3 (**Table S4**). The DEG expressions among the three subclasses were shown by a heatmap (**Figure 4A**). Genes with significant expression differences in all three possible comparisons were considered as subclass-specific genes. After merging, 263 subclass-specific genes were acquired (**Figure 4B**). GO analysis showed that the subclass-specific genes were mostly enriched in immune-related pathways, which suggested that the three subclasses may have different immune signatures (**Figure 4C**).

### Correlation of the CRC Subclasses With Immune Infiltration

**Figure 5:**
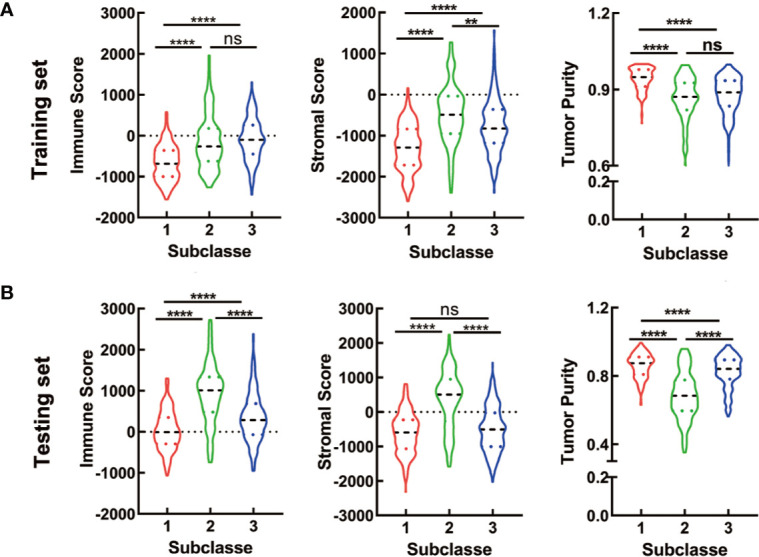
ESTIMATE analyses in the CRC subclasses. **(A, B)** The violin plot of immune score, stromal score and tumor purity from ESTIMATE of three subclasses in training set **(A)** and testing set **(B)**. For violin plots, the three lines within the boxes represent the 25th percentile, median value and the 75th percentile, respectively. The bottom and top of the plots represent the min and max value. **P < 0.01, ****P < 0.0001; ns, no significance.

To initially evaluate the tumor heterogeneity among these three subtypes, ESTIMATE algorithm was used to calculate the stromal score, immune score and tumor purity both in training and testing sets. Results showed that the three subtypes had significantly different stromal score, immune score and tumor purity (**Figure 5**). The C2 has the highest stromal score and the lowest tumor purity in training and testing sets. The immune scores of the C2 and C3 were relatively higher than that of the C1 in training set, while there was no significant difference between the C2 and C3. For testing set, the C2 has the highest immune score, which was a litter different from the training set (**Figures 5A, B** **)**.

**Figure 6:**
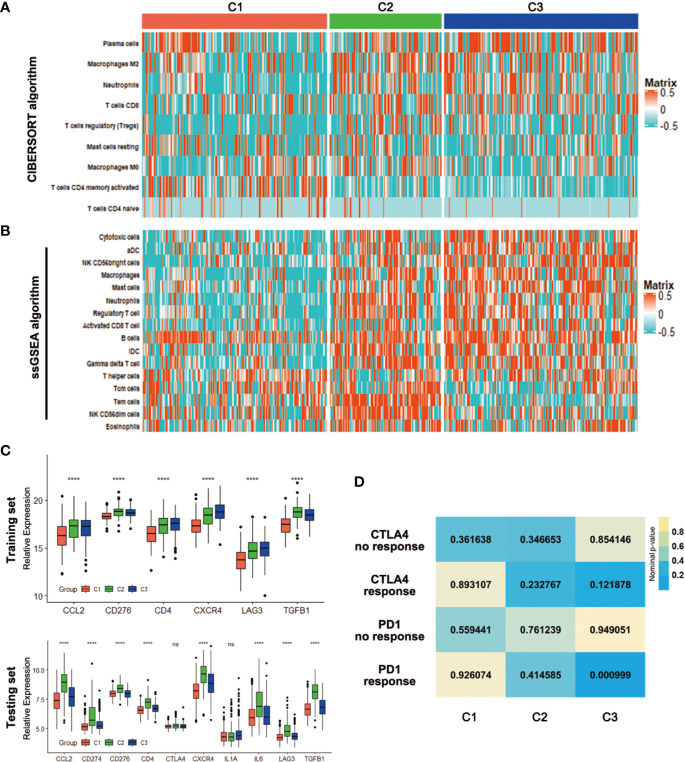
Association with immune signatures among the CRC subclasses. **(A, B)** Heatmap describing the abundance of immune cell populations in C1, C2, and C3 by CIBERSORT **(A)** and ssGSEA algorithms **(B)**. **(C)** Expression differences of several immune checkpoint genes among three subclasses in training and testing sets. **(D)** SubMap analysis for immunotherapy prediction in training set. ****P < 0.0001; ns, no significance.

With the significant difference in immune score identified among subclasses, immune infiltration was investigated to characterize their immunologic landscape. Firstly, the CIBERSORT algorithm was performed to show the differences of the LM22 gene signature within the three subtypes. There were 9 kinds of immune cell populations significantly differently enriched in the three subtypes. Plasma cells, Macrophages M2, Neutrophils and T cells CD8 were enriched in the C3 while T cells regulatory (Tregs) and Macrophages M0 were enriched in the C2, T cells CD4 memory activated and T cells CD4 naïve in the C1 (**Figure 6A**). Based on an additionally signature of 17 immune cell type (**Table S5**), more kinds of immune cells were analyzed by ssGSEA algorithm. The heatmap showed that the C2 and C3 were enriched with more immune cells, which was consistent with the result that the two had higher immune scores (**Figure 6B**). We further investigated the association between subclasses and the expression of several potentially targetable immune checkpoint genes. In training set, the expressions of checkpoint gene CCL2, CD276, CD4, CXCR4, LAG3, and TGFB1 were analyzed. The C2 and C3, especially C2, exhibited higher expression for the above immune checkpoint genes (**Figure 6C**). In testing set, additional checkpoint gene CD274, CTLA4, IL1A, and IL6 were also tested. The results coincided with that in training set, except for no significant differences with CTLA4 and IL1A (**Figure 6C**).

Considering the difference in immune infiltration patterns and expression levels of immune checkpoint genes among CRC subclasses, the probability of responding to immunotherapy was investigated by subclass mapping. We compared the expression profiles of three CRC subclasses with a published dataset (25), which included a number of 47 melanoma patients that received programmed cell death protein-1 (PD-1) immune checkpoint inhibitor or cytotoxic T-lymphocyte-associated protein-4 (CTLA-4) immune checkpoint inhibitor treatment. Data showed that the expression profile of C3 group has significant correlation with PD-1-response group (P = 0.000999), indicating that patients within C3 group were promising to respond to anti-PD-1 therapy (**Figure 6D**).

### Correlation of the CRC Subclasses With Mutations

**Figure 7:**
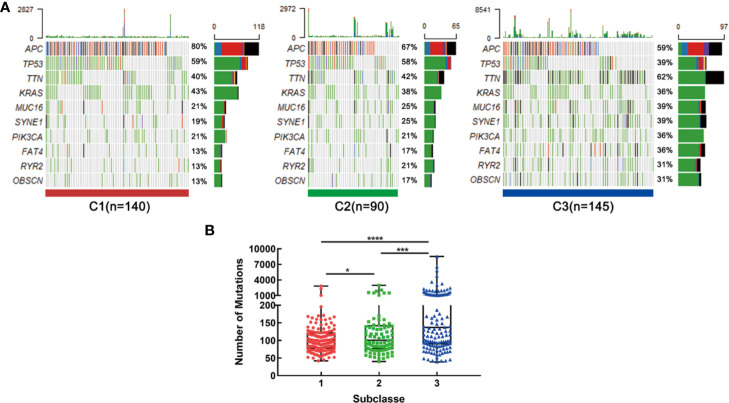
Association with mutation alterations among the CRC subclasses in training set. **(A)** OncoPrint of mutation status of top 10 genes in C1, C2, and C3. **(B)** The number of mutations in three subtypes. *P < 0.05, ***P < 0.001, ****P < 0.0001.

Recent studies have linked the gene mutations with metabolism phenotype (30). We further explored the difference of gene mutations among these three subtypes. The genes with high mutation frequency in CRC, such as APC, TP53, TTN and KRAS were examined. Results showed that distinct subclasses tended to have different mutation proportion of each gene. For example, 80 percent samples in C1 had APC mutation while only 67% in C2 and 59 percent in C3 (**Figure 7A**). What’s more, the C3 subtype had the most mutation numbers (**Figure 7B**). These data could protect samples in different clusters from choosing resistant chemotherapeutic drugs.

### Development and Validation of a Metabolism-Related Signature Using LASSO Regression Model

**Figure 8:**
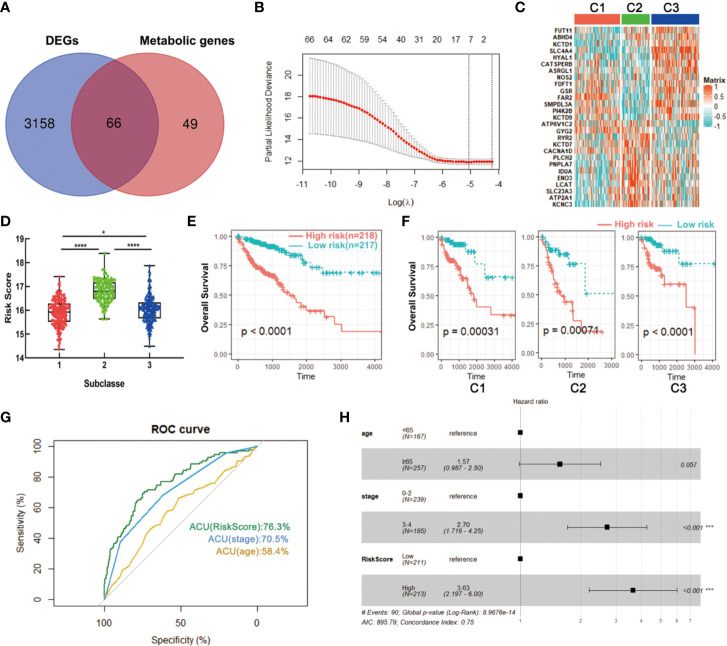
Identification of a metabolism-related signature by LASSO regression model. **(A)** Venn diagram of DEGs among three subclasses which are prognosis-related metabolic genes. **(B)** Cross-validation for tuning parameter selection in the proportional hazards model. **(C)** Heatmap of the expression levels of signature genes. **(D)** Distribution of risk scores in CRC subclasses. **(E, F)** Survival analysis of the metabolism-related signature in CRC or CRC subclasses. **(G)** ROC curve analysis of age, stage and risk score. **(H)** Multivariate Cox regression analysis of age, stage and risk score. *P < 0.05, ***P < 0.001, ****P < 0.0001. AUC, area under the curve.

To build a signature for clinical use, it is necessary to select the most representative genes of each subclass. The above data showed that a total of 3,244 DEGs were obtained among three subclasses in the training set, wherein 66 genes were significantly correlated with patients’ overall survival and had been used for metabolism-associated clustering (**Figure 8A**). Then, we applied the LASSO penalized Cox regression to identify a signature with best prognostic value (**Figure 8B**). A twenty-seven gene metabolic signature was obtained and the expression profile was distinct in three subclasses (**Figure 8C**). Furthermore, the risk scores of the metabolism-related signature were calculated with the regression coefficients (**Table S6**). The subtype C2 has the highest scores while C1 had the lowest (**Figure 8D**). Survival analysis revealed that high scores exhibited significantly poorer prognosis of CRC patients or each metabolism-associated subtype in training set (**Figures 8E, F** **)**. The results coincided with the above data that C2 had the worst prognosis. To further explore the prognostic accuracy of our signature, we performed ROC analysis to compare AUC with other factors (age and stage). It showed that the AUC of metabolism-related signature was 76.3%, higher than that of age and stage (**Figure 8G**). In addition, multivariate Cox regression analysis also confirmed the independent prognostic value of this signature (**Figure 8H**). We further applied this signature into testing set and found consistent results. Data showed that C2 has the highest scores and CRC patients with high scores had poorer prognosis (**Figures S3A, B** **)**. These data demonstrated the superior performance of metabolism-related signature for prognosis prediction, highlighting the importance of the metabolism in determining survival of CRC.

## Discussion

With the revealing of the heterogeneity in CRC, traditional paradigm of precision medicine, “one gene, one drug”, has gradually translated to “multi-gene, multi-drug” model. Better characterization of the transcriptomic subtypes, stromal and immune components for CRC may help to improve the “multi-molecular” perspective for more precise therapies (10, 31). Here, we presented a comprehensive classification of metabolism profile of CRC samples. Our results showed that CRC could be classified into three distinct metabolism-relevant subtypes, and the reproducibility of this subtyping was validated in testing set. Each subtype was associated with different clinical traits, molecular features, functions, immune cell fractions as well as gene mutation alterations.

In detail, results showed that C2 had the most kinds of differential metabolic pathways, almost all of which were downregulated in this subtype. Therefore, we defined C2 as metabolic exhausted subtype. Inversely, the majority of the metabolic pathways were enriched in C1, thus defining as metabolic active subtype. At the same time, C3 displayed intermediate metabolic activity. Our classification in CRC coincided with that in HCC, a work published recently (16). Clinical feature analyses showed that most samples in C2 were in advanced pathological stage. CRC progression signatures, such as PI3K-AKT, WNT, were also enriched in C2. Moreover, tumor microenvironment relevant estimation demonstrated that C2 had the higher immune score, stromal score and the lowest tumor purity. These data suggested that C2 subclass was of high heterogeneity and might be refractory. Our opinion was also consistent with the results that metabolic exhausted subtype C2 had the worst prognosis in both training and testing sets. The C2 subtype was somewhat similar to CMS4, one of the reported consensus molecular subtypes in CRC. CMS4 CRCs are mesenchymal prominent and characterized by activation of pathways related to epithelial–mesenchymal transition (EMT) and stemness (9). Consistently, ECM and NOTCH pathways were upregulated in our subtyped C2. Moreover, CMS4 CRCs exhibit a worse relapse-free and overall survival. A variety of immune cells were filled in C2, and C2 was presented with higher expressions of immune checkpoint genes, especially for CCL2, CD274 (also known as PD-L1), CD276, CD4, CXCR4, and TGFB, demonstrating a probably drug sensitivity toward PD-L1 antibodies (such as Nivolumab, Durvalumab) and other promising checkpoint inhibitors (32).

Compared with C2, C1, and C3 were more active in metabolism. Some key genes participated in glucose, fatty acid and glutamine metabolic process were upregulated in these two subtypes, which could be potential treatment targets. Results further revealed that C3 had the most number of mutations and accounted for the highest proportion of TTN, MUC16, SYNE1, PIK3CA, FAT4, RYR2, and OBSCN mutation compared with C1 and C2. Usually, approximately 15%–20% of CRCs harbored activating mutations in PIK3CA (33), but the mutant proportion of PIK3CA for C2 was almost doubled. As we were known, gene mutation may induce treatment resistance. A study proved that the PIK3CA mutations may potentially contribute to acquired cetuximab resistance in patients with metastatic CRC (34). Therefore, combining a PIK3CA inhibitor with an anti-EGFR antibody in the treatment of C3 subtype was recommended. What’s more, our data also has demonstrated that patients in C3 subtype might be promising to respond to anti-PD-1 therapy.

At the end of our study, we developed a metabolism-related signature that had better performance for prognosis prediction in CRC. The signature was consisted of 27 metabolic genes, which were not only expressed differentially among the three CRC subtypes but also significantly correlated with patients’ overall survival in CRC. Tumors with high risk-score displayed significantly poor prognosis in both training and testing sets.

So far, the present study was a pioneer work for CRC classification based on metabolism signature. However, we have to mention some flaws in the present study. Firstly, datasets of larger sample size are urgently needed to verify our classification. Then, the validation of our classification in clinical samples is necessary. Moreover, basic experiments are important to understand the mechanism differences among the three metabolism-relevant subtypes in CRC.

Overall, our works deepened the understanding of metabolic hallmarks of CRC, and provided valuable information for “multi-molecular” based personalized therapies and prognosis prediction.

## Data Availability Statement

Publicly available datasets were analyzed in this study. This data can be found here: TCGA data extracted from GDC data portal (https://portal.gdc.cancer.gov/), GEO (https://www.ncbi.nlm.nih.gov/geo) (GSE39582).

## Author Contributions

QZ designed and conceived the study. MZ analyzed data and drafted the manuscript. H-ZW completed and revised the manuscript. R-YP, FX, and FW provided advice and technical assistance. All authors contributed to the article and approved the submitted version.

## Funding

This study was supported by the National Natural Science Foundation of China (QZ, No. 81870390); Natural Science Foundation of Hubei Province (QZ, No. 2016CFA101).

## Conflict of Interest

The authors declare that the research was conducted in the absence of any commercial or financial relationships that could be construed as a potential conflict of interest.

## Abbreviations

CRC, Colorectal cancer; CIN, Chromosomal instability; MSI, Microsatellite instability; CIMP, CpG island methylator phenotype; CMS, Consensus molecular subtypes; HCC, Hepatocellular carcinoma; DEG, Differentially expressed gene; NMF, Non-negative matrix factorization; MAD, Median absolute deviation; OS, Overall survival; RFS, Relapse free survival; PCA, Principal components analysis; GO, gene ontology; KEGG, Kyoto Encyclopedia of Genes and Genomes; GSVA, Gene set variation analysis.
