# Supplementary material for: Metabolism-Associated Molecular Classification of Colorectal Cancer
Source: Front Oncol. 2020 Dec 4;10:602498. doi: 10.3389/fonc.2020.602498 (PMC7746835; doi:10.3389/fonc.2020.602498)
Supplement: Supplementary file 1 [file DataSheet_1.docx]

**Supplementary Figures**


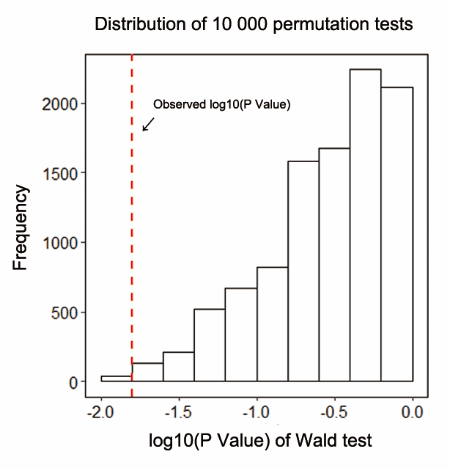


**Figure S1. Distribution of 10 000 permutation results for 115 genes in training set.**


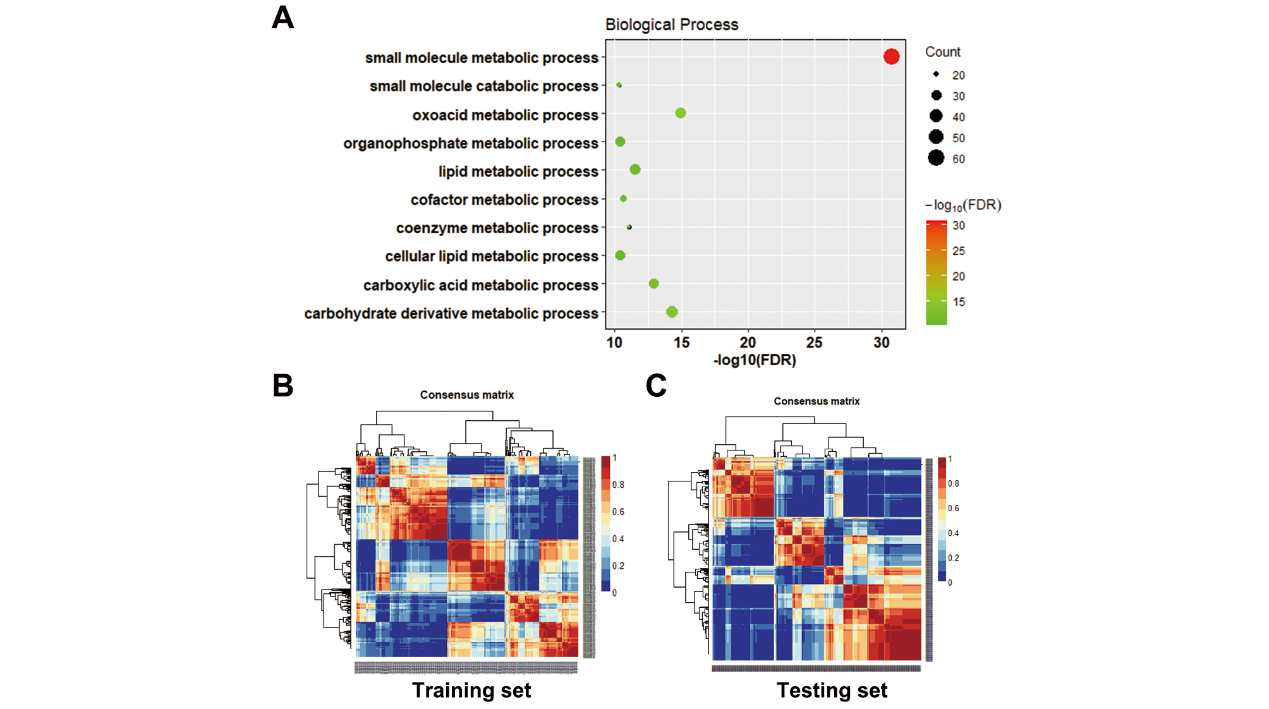


**Figure S2. Identification of CRC subclasses using NMF consensus clustering. (A)** GO analysis of 115 metabolism-associated genes. **(B, C)** The heatmap of consensus matrix when k = 3 in training set **(B)** and testing set **(C)**. GO: gene ontology.


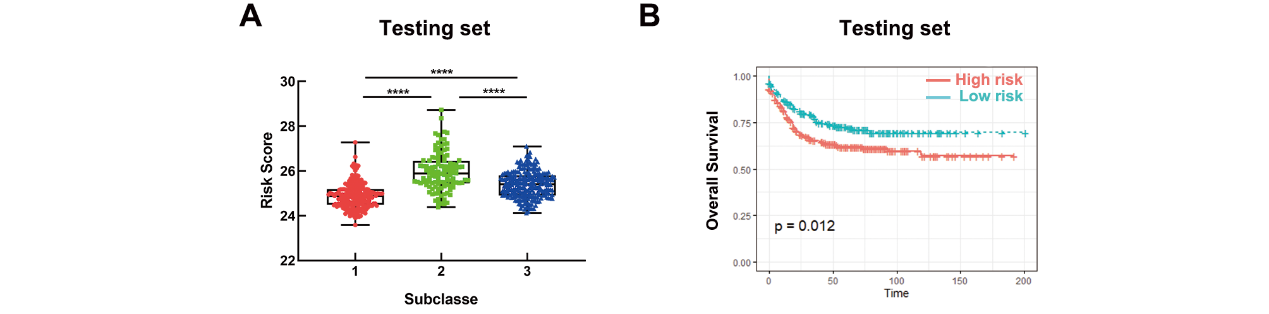


**Figure S3. Validation of the metabolism-related signature** **in testing set. (A)** Distribution of risk scores in CRC subclasses of testing set. **(B)** Survival analysis of the metabolism-related signature in CRC subclasses of testing set. ****P<0.0001.
