# Supplementary material for: Metabolism-Associated Molecular Classification of Colorectal Cancer
Source: Front Oncol. 2020 Dec 4;10:602498. doi: 10.3389/fonc.2020.602498 (PMC7746835; doi:10.3389/fonc.2020.602498)
Supplement: Supplementary file 2 [file DataSheet_2.docx]

**Supplementary methods**

**Distribution of 10 000 re-sampling Wald tests in training set with the 115 metabolic genes for classification**

The expression matrix of the 115 selected genes were extracted from the training set. Their expression data were summarized to build up a compound score for each patient. The compound score was used as the independent variable to perform OS analysis based on the Cox model. The Wald test P value was saved as the observed P value. For the re-sampling test, we randomly chose 115 genes from a total of 1 238 initially filtered metabolic genes. We repeated the re-sampling and survival analysis procedure for 10 000 times, obtaining 10 000 re-sampling Wald test P values. Then, the observed and re-sampling P values were transformed into log10 format, and we plotted a histogram of the 10 000 re-sampling log10 (P values). The observed log10 (P value) was added.

**NMF consensus clustering**

After sample filtration and gene filtration, a 115 metabolic-related gene expression matrix was obtained for classification. NMF clustering was implemented by “NMF” R package in training and testing sets, and the codes were provided as follow:

library(NMF)

data2<- read.csv (file = "TCGA-metabolic115-Ex.csv", row.names=1, header=T, check.names=F)

dim(data2)

class(data2)

data<- as.matrix(data2)

estim.r <- nmf(data, 2:6, nrun=20, seed=123456)

plot(estim.r)

coph <- estim.r$measures$cophenetic

plot(2:6,coph, type="b", col="purple",xlab="", ylab="",lwd=3)

title(xlab="Factorization rank", ylab="Cophenetic coefficient", cex.lab=1)

coph_diff <- NULL

for (i in 2:length(coph))

{ coph_diff <- c(coph_diff, coph[i-1]-coph[i]) }

k.best <- which.max(coph_diff)+1

k.best

g<-predict(estim.r[["fit"]][["3"]])

c<- as.data.frame(g)

write.csv(c,"g-3-RUN20.csv")

map<- estim.r[["consensus"]][["3"]]

consensusmap(map)
